# Raman Spectroscopic Signature of Ectoine Conformations in Bulk Solution and Crystalline State

**DOI:** 10.1002/cphc.202000457

**Published:** 2020-08-17

**Authors:** Tihomir Solomun, Marc Benjamin Hahn, Jens Smiatek

**Affiliations:** ^1^ Bundesanstalt für Materialforschung und -prüfung (BAM) 12205 Berlin Germany; ^2^ Freie Universität Berlin Institut für Experimentalphysik 14195 Berlin Germany; ^3^ Institut für Computerphysik Universität Stuttgart 70569 Stuttgart Germany

**Keywords:** Crystals, DFT Calculations, Ectoine, Osmolyte Hydration, Raman Spectroscopy

## Abstract

Recent crystallographic results revealed conformational changes of zwitterionic ectoine upon hydration. By means of confocal Raman spectroscopy and density functional theory calculations, we present a detailed study of this transformation process as part of a Fermi resonance analysis. The corresponding findings highlight that all resonant couplings are lifted upon exposure to water vapor as a consequence of molecular binding processes. The importance of the involved molecular groups for water binding and conformational changes upon hydration is discussed. Our approach further shows that the underlying rapid process can be reversed by carbon dioxide saturated atmospheres. For the first time, we also confirm that the conformational state of ectoine in aqueous bulk solution coincides with crystalline ectoine in its dihydrate state, thereby highlighting the important role of a few bound water molecules.

## Graphical abstract

**Spectroscopic Signature**: By means of confocal Raman spectroscopy and density functional theory calculations, we present a detailed study of conformational changes for zwitterionic ectoine upon hydration as part of a Fermi resonance analysis. The corresponding findings highlight that all resonant couplings are lifted upon exposure to water vapor as a consequence of molecular binding processes. Our approach further shows that the underlying rapid process can be reversed by carbon dioxide saturated atmospheres. For the first time, we also confirm that the conformational state of ectoine in aqueous bulk solution coincides with crystalline ectoine in its dihydrate state, thereby highlighting the important role of a few bound water molecules. The TOC figure shows the corresponding Raman spectra of ectoine with equatorial and axial carboxylate group conformation. The transformation process between equatorial and axial conformations upon hydration can be reversed by addition of carbon dioxide.

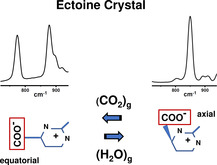

## 1 Introduction

Ectoine (2‐Methyl‐1,4,5,6‐tetrahydropyrimidine‐4‐carboxylic acid) is a zwitterionic, hygroscopic, low‐weight organic molecule which is produced by extremophilic bacteriae in presence of harsh environmental conditions like high salinity.^1^, ^2^ Recent experimental and computational results revealed a broad plethora of interesting effects for ectoine in aqueous bulk solution. The presence of strongly bound water molecules around ectoine and the corresponding effects on the water structure were discussed as a rationale for the stabilizing and protective effects on proteins and lipid bilayers.^3^, ^4^, ^5^ In addition to this remarkable water binding behaviour and the resulting hygroscopicity,^2^ it was assumed that the properties of the first hydration shell around ectoine are responsible for further effects, ranging from the stabilization of proteins^1^, ^6^, ^7^, ^8^, ^9^, ^10^ to the protection of DNA from ultraviolet^11^ and ionizing radiation damage.^12^, ^13^ In contrast to its stabilizing effects, previous studies also reported a destabilizing impact of ectoine on charged macromolecules with regard to direct and local interactions^11^, ^14^, ^15^, ^16^, ^17^, ^18^, ^19^ in addition to radical scavenging properties.^11^, ^12^, ^20^

**Figure 1:**
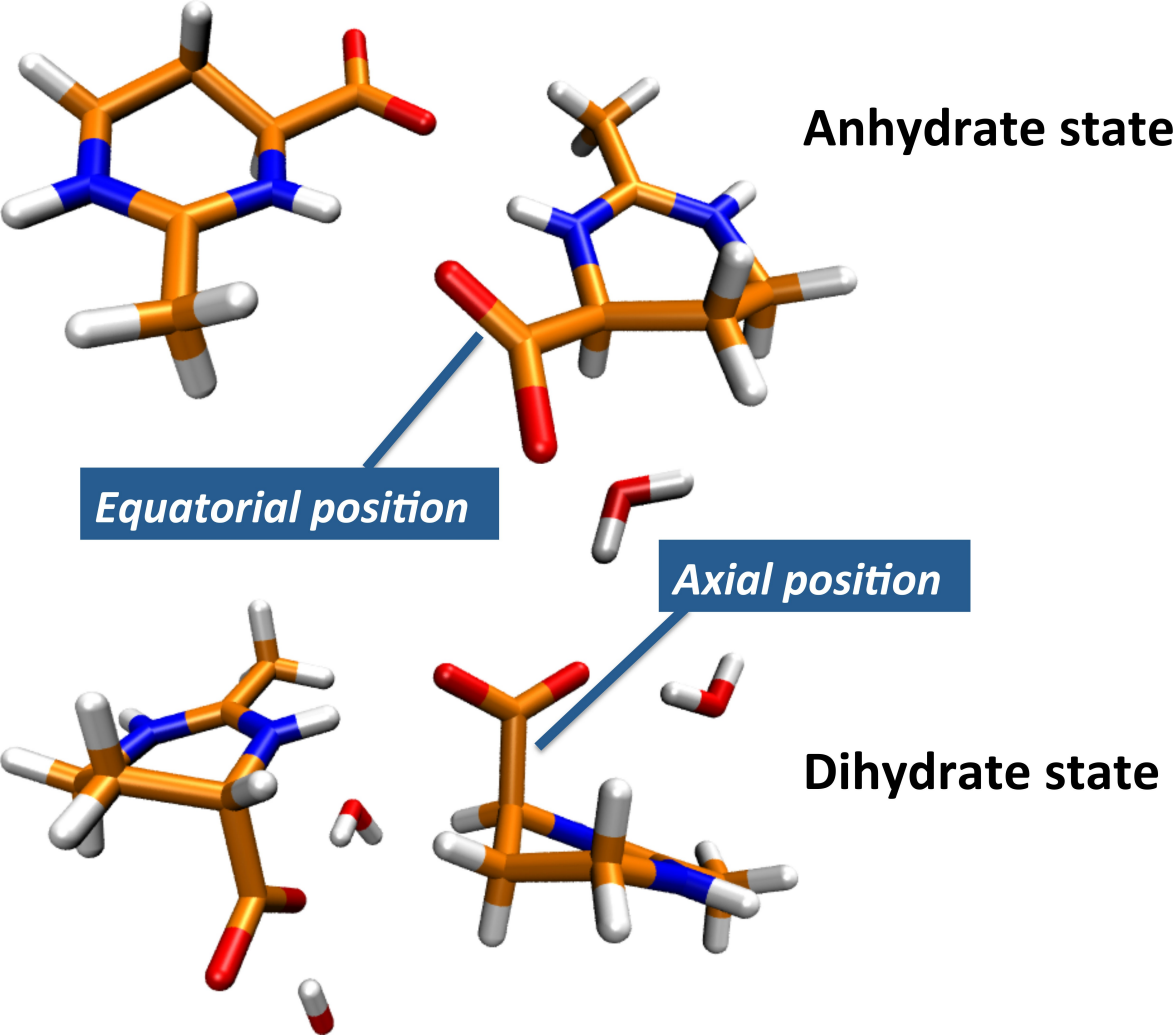
Top: Anhydrate form of two ectoine molecules with the carboxylate group in equatorial position. Bottom: Dihydrate form of two ectoine molecules with the surrounding water molecules in contact with the carboxylate group in axial position. Blue colors denote nitrogen atoms, orange colors mark carbon atoms, hydrogen atoms are colored in white and all oxygen atoms in red color.

Besides the aforementioned effects in bulk solution, recent single‐crystal and powder X‐ray, as well as single crystal neutron diffraction measurements also revealed conformational changes of crystalline ectoine upon dehydration.^21^ In more detail, ectoine in its crystalline dihydrate state forms nanometer‐sized channels with bound water molecules. Over a few days and even at ambient conditions, the dihydrate state undergoes a loss of water and finally transforms into a highly hygroscopic anhydrate form (Figure 1). This transition also involves a significant conformational change of the carboxylate group from axial position into an energetically more favorable equatorial conformation. It was shown by density functional theory (DFT) calculations, that the corresponding changes in the conformations and the associated metastable and stable states are strongly influenced by the amount of hydrating water molecules.^21^

In this article, we study the transformational change of solid ectoine upon hydration and dehydration by means of confocal Raman spectroscopy and DFT calculations. The strong binding of water molecules to ectoine provides unique conditions for spectroscopic analyses as part of vibrational studies. The associated vibrational coupling and Fermi resonances allow us to monitor and to study in full detail the crucial interactions with water molecules. With regard to these points, our approach thus extends previous experimental studies^21^ and elucidates the strong interactions between water and ectoine in combination with certain consequences for conformational changes. Our findings are then applied to the situation of ectoine in bulk water which shows by comparison a conformational agreement with crystalline ectoine in its dihydrate form. These results demonstrate that only a few bound water molecules are necessary to switch between different conformations as well as stable and metastable states.

## 2 Theoretical Background: Fermi Resonances

Fermi resonance is a very common mechanism in vibrational spectra of polyatomic molecules with complex structure.^22^ It appears when a fundamental vibrational frequency lies closely to an overtone or combination frequency. Usually these vibrations concern the same part of the molecule and necessarily belong to the same symmetry point group such that any interaction with further molecules leads to their disappearance. As a result of the Fermi resonance, two peaks can be observed in the spectra so that the energy is transferred between the two frequencies. Most often, one mode is increased in its magnitude whereas the other one is decreased. According to the treatment of Betran *et al*.^23^ and Devendorf *et al*.,^24^ the frequency gap Δ between an observed Fermi resonance doublet reads

$$\Delta =\sqrt{\Delta_{0}^{2}-4W^{2}}$$

with the unperturbed frequency spacing $\Delta_{0}$ and the anharmonic coupling strength W . Furthermore, the coupling strength W is calculated from the experimentally determined spectrum via the intensity ratio R according to

$$R=\frac{I_{a}}{I_{b}}=\frac{\Delta +\sqrt{\Delta^{2}-4W^{2}}}{\Delta -\sqrt{\Delta^{2}-4W^{2}}}$$

where $I_{a}$ and $I_{b}$ are the observed peak intensities (or integrated peak areas) of the Fermi doublet. It has been shown by Placzek^25^ that the intensity ratio can be approximated by

$$R\approx \frac{2W-\Delta }{2W+\Delta }$$

when ignoring all prefactors.

## Experimental and Numerical Details

Anhydrate crystalline ectoine powder with >95% purity was purchased from Sigma‐Aldrich. Ultra pure water (LiChrosolv for chromatography) and heavy water (Uvasol, for NMR spectroscopy with deuteration degree >99.99% ) were obtained from Merck (Germany). Confocal Raman measurements were performed with a confocal Alpha300R instrument (WITec, Germany), equipped with a 20x Zeiss EX Epiplan DIC objective, a 532 nm laser (Excelsior 532–60) with a laser power of 20 mW. The spectrometer was an UHTS‐300‐VIS (grid of 600 gratings/mm) and an thermoelectrically cooled CCD‐camera Andor DV‐401A‐BV‐532 at −64 °C. The spectra were obtained through high precision Zeiss cover glasses focusing on one of the ectoine crystals. A picture of the crystal is shown in the supporting material. After obtaining adequate and optimized spectra of anhydrate ectoine, the sample was covered with a small vessel containing a few drops of the respective solvent or some CO_2_ crystals to rapidly form a fully saturated H_2_O, D_2_O or CO_2_ atmosphere, respectively. A series of spectral measurements at 4 s time‐resolution (integration time 3 s, measurements interval 1 s) were obtained. The anhydrate‐to‐dihydrate ectoine transformation under the influence of water vapour took a few minutes to start and additionally few tens of seconds to reach the final stable hydration state. During this time, the solvent molecules (D_2_O or H_2_O) penetrated the probed confocal volume, expected to be few microns under present experimental conditions.^26^ The reverse dihydrate‐to‐anhydrate transformation in CO_2_ atmosphere took somewhat longer, about few tens of minutes to produce fully anhydrated ectoine within the confocal volume.

### DFT calculations

In accordance with previous publications,^5^, ^14^ all DFT calculations were performed with the software package Orca 4.0.0.2^27^, ^28^ and with the generalized gradient approximation BLYP functional.^29^, ^30^ The Kohn‐Sham orbitals were expanded into the def2‐TZVPP basis set^31^ with dispersion corrections^32^ and with the Becke‐Johnson damping scheme.^33^ For the calculation of the spectra with the method described in Ref. [34], we used an identical approach like in Ref. [14] where ectoine is interacting with 4 water molecules. In addition, we also calculated the spectrum of a single zwitterionic ectoine molecule embedded by a continuum solvent with a dielectric constant $\epsilon_{r}=80.4$ . In addition, ground‐state energies were calculated for single zwitterionic ectoine molecules with axial as well as equatorial carboxylate group conformations, both in continuum solvent^35^ and in gas phase approximation. Before the calculation of spectra, a minimization of the total energy (geometry optimization) was performed for 100 cycles.

## 3 Results and Discussion

**Figure 2:**
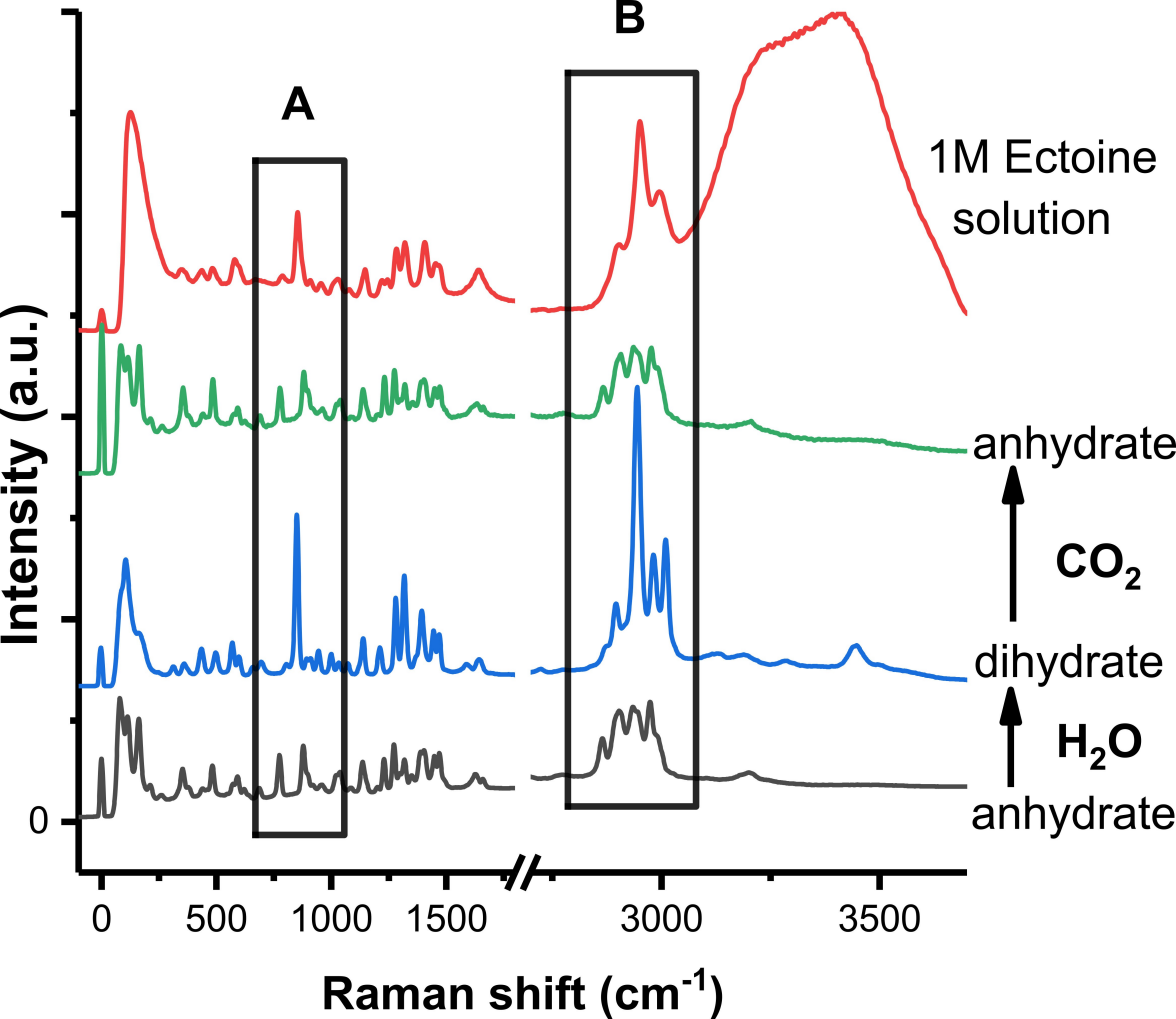
Confocal Raman spectra of anhydrated and dihydrated solid ectoine and in 1 M ectoine aqueous solution (red). The consecutively obtained spectra concerned pristine anhydrate ectoine (black), ectoine exposed to H_2_O atmosphere (blue), followed by exposure to CO_2_ atmosphere (green). The spectra are normalised to the band at 1140 cm^−1^ which is known to be relatively insensitive to the ectoine hydration state. The two spectral ranges of interest here are marked by rectangles: A) the Fermi resonance of the anhydrate ectoine ring deformations and ring breathing modes with the doublet observed at 775 cm^−1^ and 878 cm^−1^, and dihydrate ectoine with the single band of the ring breathing mode at 852 cm^−1^. B) the Fermi resonance of the methyl group of the anhydrate with the doublet observed at 2934 cm^−1^ and 2970 cm^−1^, concerning coupling of a C−H bending overtone and C−H stretching, the stretching in ectoine dihydrate is observed as a narrow predominant band at 2943 cm^−1^. See text for details.

Representative confocal Raman spectra of anhydrated and dihydrated ectoine and in aqueous 1 M ectoine bulk solution are shown in Figure 2. Two important anharmonic couplings in the regions 750 cm^−1^ to 900 cm^−1^ and 2900 cm^−1^ to 3000 cm^−1^ can be identified. These frequencies correspond to the two Fermi resonances, one involving ring deformation modes and the other the methyl group of ectoine, respectively. A visualization of these vibrations is shown in the supporting material. As can be seen, these two spectral features can be used to distinguish anhydrated from dihydrated solid ectoine in combination with the two conformations of the carboxylate group (Figure 1) as discussed in Ref.[21]. Furthermore, the preponderance of these bands is directly related to the hydration process as will be discussed in detail in the following.

### 3.1 Fermi resonances in anhydrate ectoine

The prominent Fermi resonance of anhydrate solid ectoine (doublet located at 775 and 878 cm^−1^ in Figure 2, black A) involves the combination band of two ring deformation modes at 352 cm^−1^ and 482 cm^−1^ which form a Fermi resonance with a ring breathing mode at about 852 cm^−1^. From previous DFT calculations^14^ it is known, that the combination mode has the same point group symmetry as the ring breathing mode, thereby satisfying the necessary condition for the occurrence of a Fermi resonance as shown in the supporting material. Importantly, all involved features contain contributions from the scissor vibrational mode of the carboxylate group. According to the discussion above and by setting the doublet splitting to 2 *W*=103 cm^−1^ in combination with the peak area intensity ratio *R*≈0.869, a separation of Δ≈7 cm^−1^ is obtained for the coupling modes before Fermi resonance. This is in good agreement with the observed shift of Δ=12 cm^−1^ from 364 cm^−1^ to 352 cm^−1^ for the ring deformation mode upon hydration (supporting material). The assignment of the resonance is corroborated by the study of p‐cresol and related molecules where an observed Raman doublet reveals Fermi resonance between the symmetric ring‐breathing fundamental and the overtone mode of the non‐planar ring vibration at 413 cm^−1^.^36^

Fermi resonances also often occur in the C−H stretching region of molecules.^37^ For the methyl group of ectoine, the Fermi resonance of interest occurs in the 2900 cm^−1^ spectral region, where a coupling between the C−H symmetric stretch fundamental and a C−H bend overtone gives rise to two prominent bands (Figure 2, box B). In fact, several overtones and combination bands from the bending region are expected to be close to the CH_3_ stretching mode with the same symmetry, so that multiple resonances can be expected. Therefore, it is difficult to clearly establish which CH_3_ bending overtone (or combination of them) is involved in the Fermi resonance. However, the Fermi resonance of gaseous methanol in the C−H stretching region was assigned to bands at 2925 cm^−1^ and 2955 cm^−1[37]^ which is in reasonable agreement with the bands of anhydrated ectoine at 2934 cm^−1^ and 2970 cm^−1^ (Figure 2, box B). The disappearance of the resonances upon hydration clearly implies the important role of the CH_3_ group for water binding.

### 3.2 Monitoring ectoine hydration

**Figure 3:**
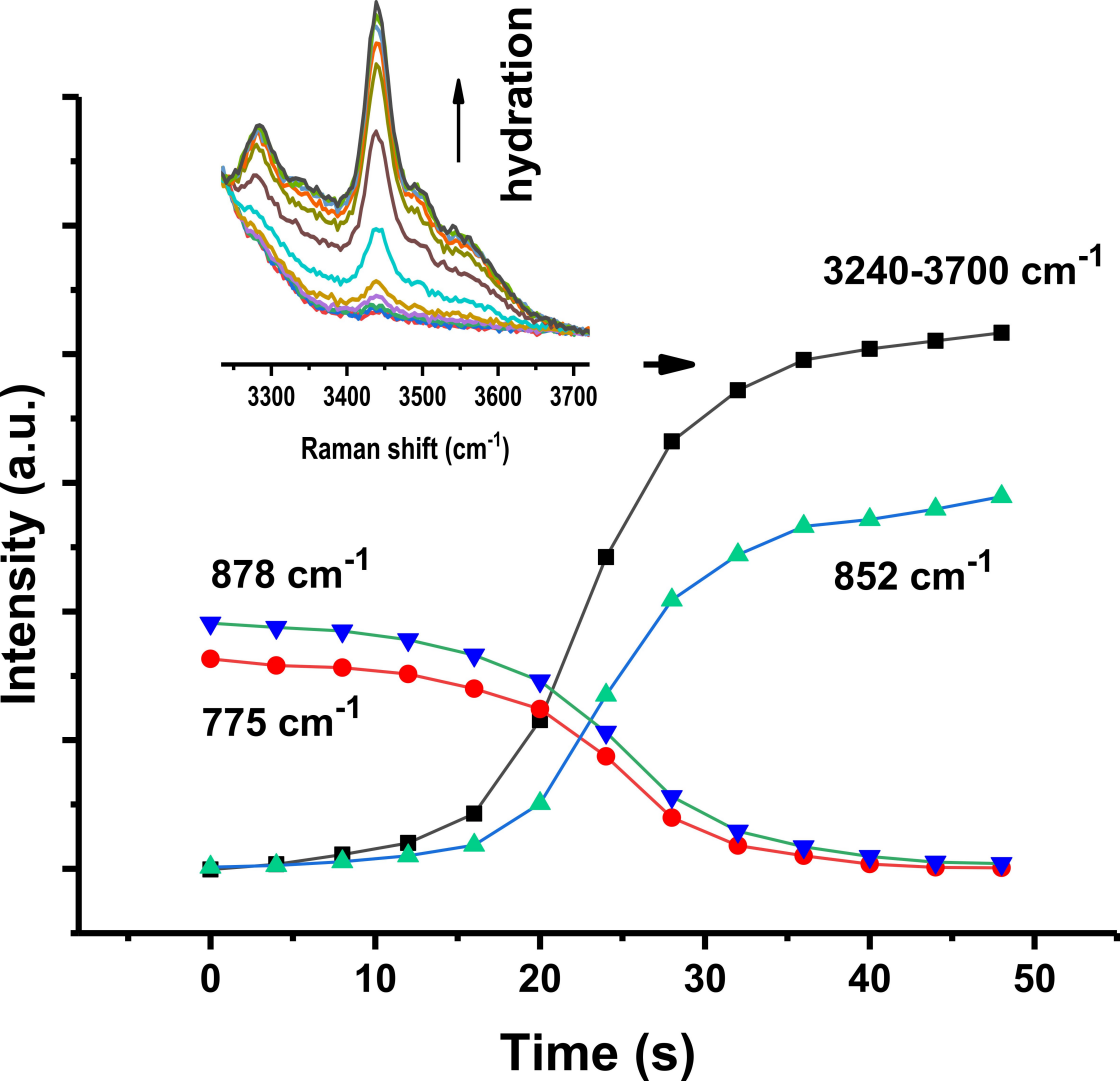
Integrated area intensities of bands (difference between dihydrate and anhydrate ectoine spectra at specified wavenumbers) as a function of exposure time to water‐saturated atmosphere. In addition to single bands, the area intensity of the water stretching region above 3240 cm^−1^ is shown in the inset. The hydration of ectoine within the confocal volume stops at the point at which the two Fermi resonance bands at 775 and 878 cm^−1^ disappear.

**Figure 4:**
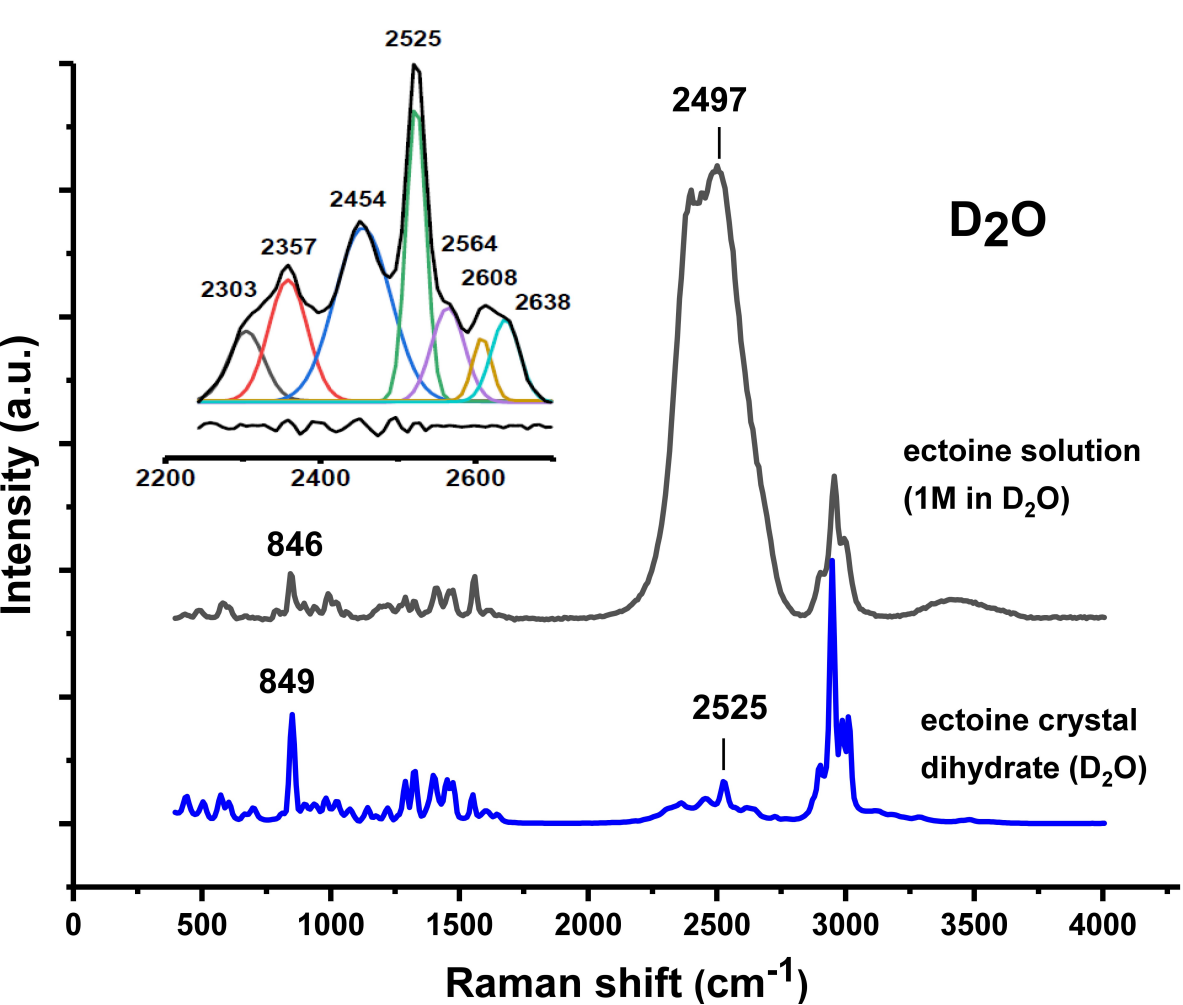
Raman spectra of solid ectoine after exposure to D_2_O saturated atmosphere and for 1 M ectoine solution in D_2_O. The inset shows the D−O stretching region around 2500 cm^−1^ of hydrated ectoine fitted with 7 bands, as well as the residual at the bottom of the inset. For clarity, the spectra are shifted vertically.

The corresponding integrated spectral densities of the aforementioned bands as a function of time are presented in Figure 3. As can be seen, all anharmonic spectral features of anhydrate ectoine quickly disappear upon exposure to water vapour. In more detail, a few minutes after exposure to water vapour, the integrated area densities for wavenumbers 775 cm^−1^ and 878 cm^−1^ (Figure 2A) start to decrease rapidly, while area intensities for 852 cm^−1^ increase (Figure 3). This behavior correlates directly with the binding of water to ectoine as revealed by an increase in the intensity of the O−H stretching region above 3240 cm^−1^ (inset of Figure 3). It is worth noting here that the O−H stretching peak of bulk water reveals a very broad band with a complex line shape which is fitted in the experimental spectrum with four Gaussian functions.^5^ The bands below 3600 cm^−1^ are attributed to the distinct contribution of the collective modes to the molecular polarizability. Thereby the intensity of the modes within distorted tetrahedral networks contribute at a higher frequency and, those involved in a ice‐like network contribute at a lower frequency. The hydration behaviour is not restricted to water, as a comparable behaviour can also be observed upon exposure of anhydrated ectoine to saturated D_2_O atmosphere (Figure 4). As can be seen a D_2_O‐ectoine complex reveals at least 7 different states in the D−O stretching region around 2500 cm^−1^. Since, there is no overlap between ectoine C−H and D−O stretching frequencies (in contrast to H_2_O at Raman shifts larger than 2800 cm^−1^ (Figure 2)), the spectrum can also be used to estimate the amount of D_2_O molecules bound to crystalline ectoine. This was achieved by the normalization of the C−H stretching region of ectoine and in comparison with the area intensities of the D−O stretching region for D_2_O hydrated ectoine with 1 M ectoine D_2_O solution (molar ratio D_2_O:ectoine=50 : 1). The corresponding calculation yields a value of 3.2 D_2_O molecules bound to one ectoine molecule within the crystal. While this number is slightly higher when compared to the recently reported results for dihydrate crystal structures with 2 water molecules per ectoine,^21^ it has to be noted that we did not include the unknown partial molar volume occupied by ectoine in 1 M solution for corrections.

Interestingly, the reverse process as represented by the rapid transformation from dihydrated to anhydrated solid ectoine, can be triggered by the exposure to CO_2_ saturated atmospheres (Figure 2, green line). Although this conformational change is slower than the hydration process, it still can be considered as a rapid exchange mechanism, such that a complete dehydration of ectoine within the considered confocal volume can be observed on a time scale of few tens of minutes. The mechanistic aspects of this reaction are not known, however, we propose by reasons of chemical intuition that the transiently formed unstable H_2_CO_3_ species within the nanometer size channels in the ectoine dihydrate crystal decompose under water removal. In the case of 1 M ectoine in aqueous bulk water solution (Figure 2, red line) a strong band near 850 cm^−1^ is observed, while the two bands at 775 cm^−1^ and 877 cm^−1^ are missing. The solution spectrum is also closely related to the dihydrate spectrum with respect to the C−H stretching region around 2950 cm^−1^ (Figure 2, box B). The excellent qualitative agreement between solid dihydrate ectoine and ectoine in bulk water implies that the carboxylate group of ectoine adopts an axial conformation in both environments. Therefore, it can be concluded that the axial conformation is stabilized under the influence of water molecules^21^ and does not change upon the formation of a full hydration shell.

### 3.3 Computed Raman spectra from DFT calculations

The differences in the electronic ground state energies $\Delta E=E\left(\mathrm{eq}\right)-E\left(\mathrm{ax}\right)$ as calculated for single ectoine molecules with the carboxylate group in axial (ax) and in equatorial (eq) conformation embedded into a continuum water model as well as in gas phase are presented in Table 1.

**Table 1:** Differences in the electronic ground state energies $\Delta E=E\left(\mathrm{eq}\right)-E\left(\mathrm{ax}\right)$ as calculated for single ectoine molecules with the carboxylate group in axial (ax) and in equatorial (eq) conformation in a continuum water model and in gas phase.

Gas phase/Continuum water	ΔE [kJ//mol]
Continuum water	−5.51
Gas phase	−10.03

**Figure 5:**
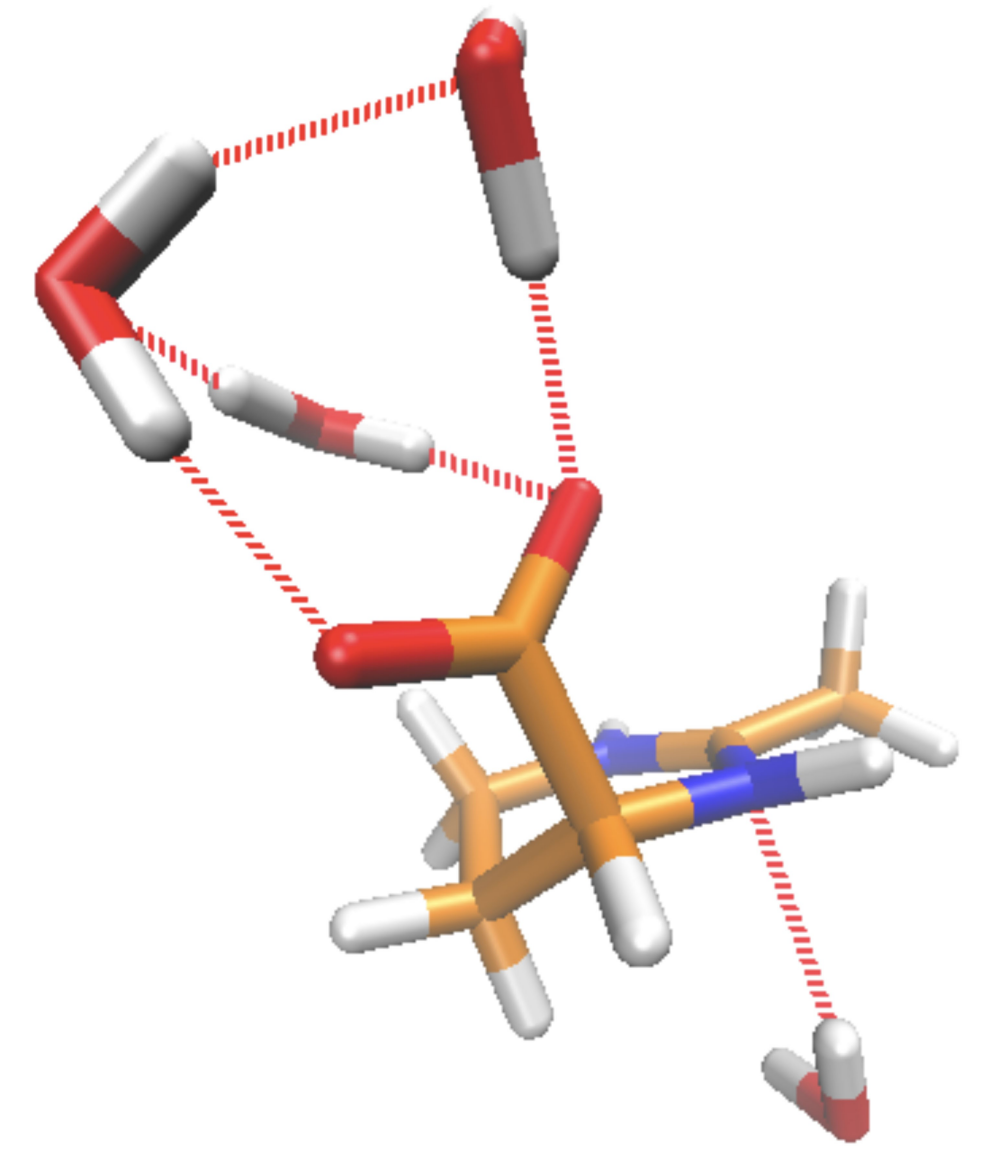
Snapshot of geometry‐optimized ectoine in contact with four water molecules. Slashed red lines denote hydrogen bonds. Blue colors denote nitrogen atoms, orange colors mark carbon atoms, hydrogen atoms are colored in white and all oxygen atoms in red color.

Despite all previous findings, the corresponding results reveal that the equatorial state is more stable than the axial conformation in both gas phase and continuum water. The respective energy differences are ΔE=-5.51 kJ/mol in continuum water and ΔE=-10.03 kJ/mol in gas phase. As we will point out in the remainder of this section, these results can be rationalized by the missing crucial contribution of explicit water molecules. Notably, the energy differences between gas phase and continuum solvent $\Delta E_{S}\left(\mathrm{ax}/\mathrm{eq}\right)=E_{\mathrm{sol}}\left(\mathrm{ax}/\mathrm{eq}\right)-E_{\mathrm{gas}}\left(\mathrm{ax}/\mathrm{eq}\right)$ reveal that the axial conformation ($\Delta E_{S}\left(\mathrm{ax}\right)=-139.86$ kJ/mol) is more stabilized than the equatorial state ($\Delta E_{S}\left(\mathrm{eq}\right)=-135.34$ kJ/mol) in presence of continuum water which highlights the strong water affinity of the axial state. In contrast to continuum solvent and gas phase approximations, the outcomes of the DFT calculations change in presence of explicit water molecules. By means of energy minimization routines in terms of geometry optimization, one can observe that ectoine surrounded by four water molecules and in presence of a continuum water dielectric background indeed prefers the axial conformation (Figure 5). Moreover, any initial equatorial conformation transforms into an axial state after a sufficient amount of geometry optimization steps which points to the fact that the equatorial state is not stable in presence of explicit water molecules. The corresponding results are in reasonable agreement with recent discussions and emphasize the crucial role of direct water binding for the conformational behavior of ectoine.^21^

**Figure 6:**
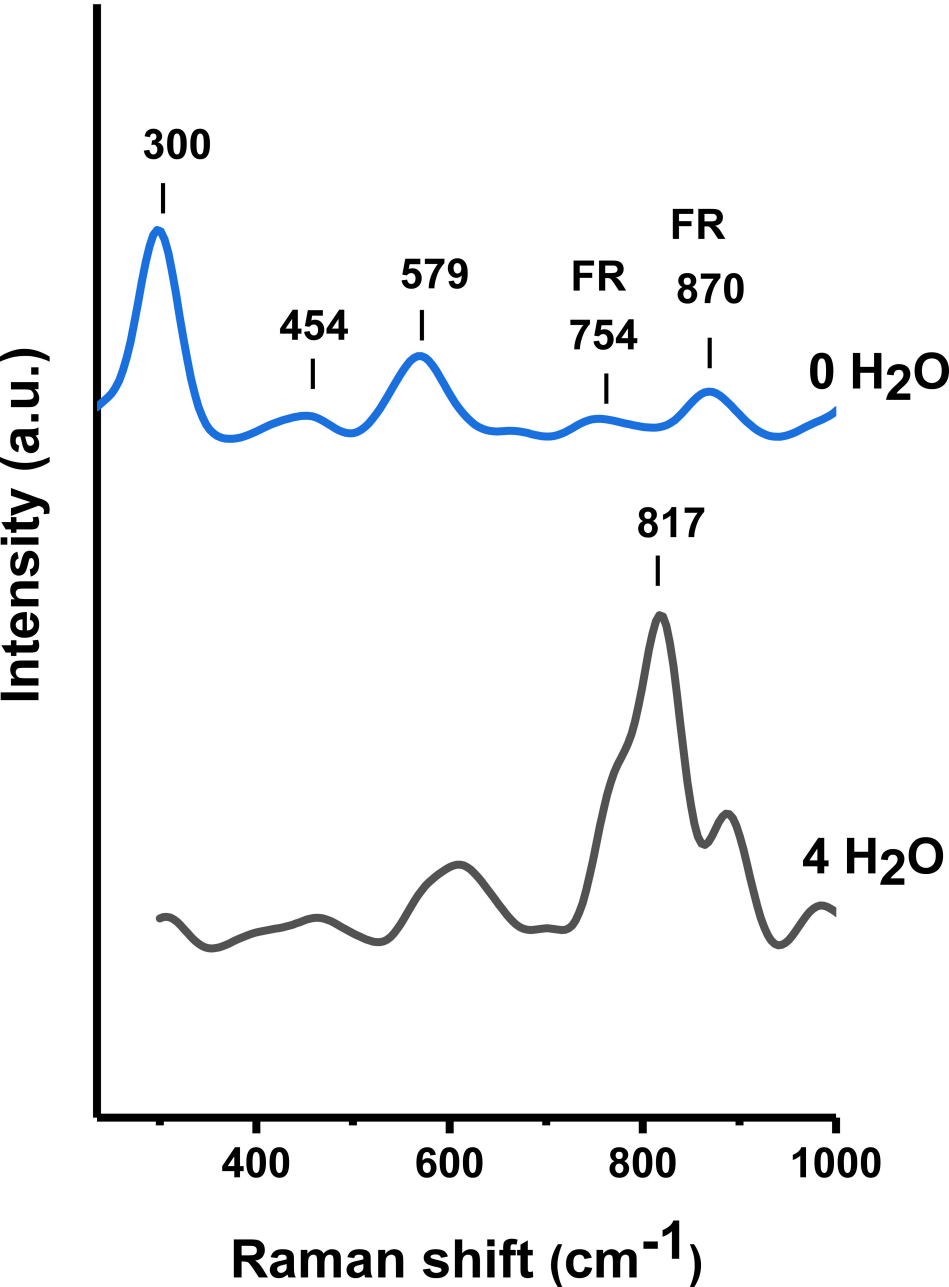
Calculated Raman spectra for continuum phase ectoine (top) and ectoine hydrated with 4 water molecules (bottom).

The computed Raman spectra for ectoine in the axial state with four water molecules as well as for single ectoine in a dielectric continuum solvent are shown in Figure 6. Both conformations reveal an axial conformation of the carboxylate group. In more detail, three water molecules strongly interact via hydrogen bonds with the carboxylate group while a fourth water molecules interacts with the ring nitrogen atom. From the fingerprint region between 250 cm^−1^ and 1050 cm^−1^ it becomes clear that a number of features in the calculated Raman spectra are consistent with the experimental results as discussed above. The presence of hydrating water molecules around ectoine implies the emergence of a strong band around 817 cm^−1^. A comparable band can also be observed in the experimental results for dihydrated ectoine at 852 cm^−1^ (Figure 2). In absence of explicit water molecules around ectoine in a continuum solvent, a strong band near 800 cm^−1^ is missing, while two medium intensity bands at 754 and 870 cm^−1^ can be observed. In agreement with the considerations above, a pronounced ring deformation mode at about 300 cm^−1^ becomes visible which borrows the intensity from the Fermi coupling. With regard to these findings, one has to conclude that the observed disappearance of Fermi resonances upon hydration requires the interaction with water molecules.

## 4 Summary and Conclusion

Confocal Raman spectroscopy measurements were used to study the vibrational behavior of anhydrated and dihydrated ectoine crystals. The spectra reveal that the vibrational spectrum of solid and anhydrated ectoine is dominated by a significant amount of anharmonic interactions. Upon exposure to a saturated water (or D_2_O) atmosphere, these interactions are rapidly lifted as a result of the formation of the ectoine‐water complex. The associated conformational changes, meaning the transition of the carboxylate group from the equatorial to an axial conformation influences the ring deformation modes which are spectroscopically characterized. We are able to monitor the conformational change as well as the hydration process which takes place on a time scale of a few tens of seconds. Our results reveal that only a few water molecules instead of a full hydration shell are required to initiate this transition. Main molecular groups to identify this process are represented by the carboxylate as well as the methyl group. Further DFT calculations highlight that the disappearance of Fermi resonances is solely attributed to the interaction with water molecules instead of any conformational changes. The juxtaposition of the present data and the literature crystallographic results permits unambiguous assignment of the solution ectoine structure with axial carboxylate group conformation. The results of this study shed more light on the crucial interaction between ectoine and water molecules. The underlying hydration process can be reversed under carbon dioxide atmosphere which highlights the subtle balance of stable and metastable conformations. One may ask if these slight changes as well as the strong water interactions are of further importance for stabilizing and destabilizing effects of ectoine in macromolecular environments.

## Conflict of interest

The authors declare no conflict of interest.

## Supporting information

As a service to our authors and readers, this journal provides supporting information supplied by the authors. Such materials are peer reviewed and may be re‐organized for online delivery, but are not copy‐edited or typeset. Technical support issues arising from supporting information (other than missing files) should be addressed to the authors.

Supplementary
