# Supplementary material for: Raman Spectroscopic Signature of Ectoine Conformations in Bulk Solution and Crystalline State
Source: Chemphyschem. 2020 Aug 17;21(17):1945–50. doi: 10.1002/cphc.202000457 (PMC7540454; doi:10.1002/cphc.202000457)
Supplement: Supplementary file 1 — Supplementary [file CPHC-21-1945-s001.pdf]

ChemPhysChem

Supporting Information

Raman Spectroscopic Signature of Ectoine Conformations in Bulk Solution and Crystalline State

Tihomir Solomun,* Marc Benjamin Hahn, and Jens Smiatek*

Author Contributions

T.S. Conceptualization:Equal; Formal analysis:Lead; Investigation:Equal; Methodology:Equal; Project administration:Lead; Validation:Equal; Writing - Original Draft:Equal; Writing - Review & Editing:Equal
M.H. Conceptualization:Supporting; Data curation:Supporting; Formal analysis:Supporting; Investigation:Supporting; Software:Supporting; Validation:Supporting; Writing - Original Draft:Supporting; Writing - Review & Editing:Supporting
J.S. Conceptualization:Equal; Data curation:Equal; Formal analysis:Equal; Investigation:Equal; Software:Lead; Supervision:Equal; Validation:Equal; Writing - Original Draft:Equal; Writing - Review & Editing:Equal

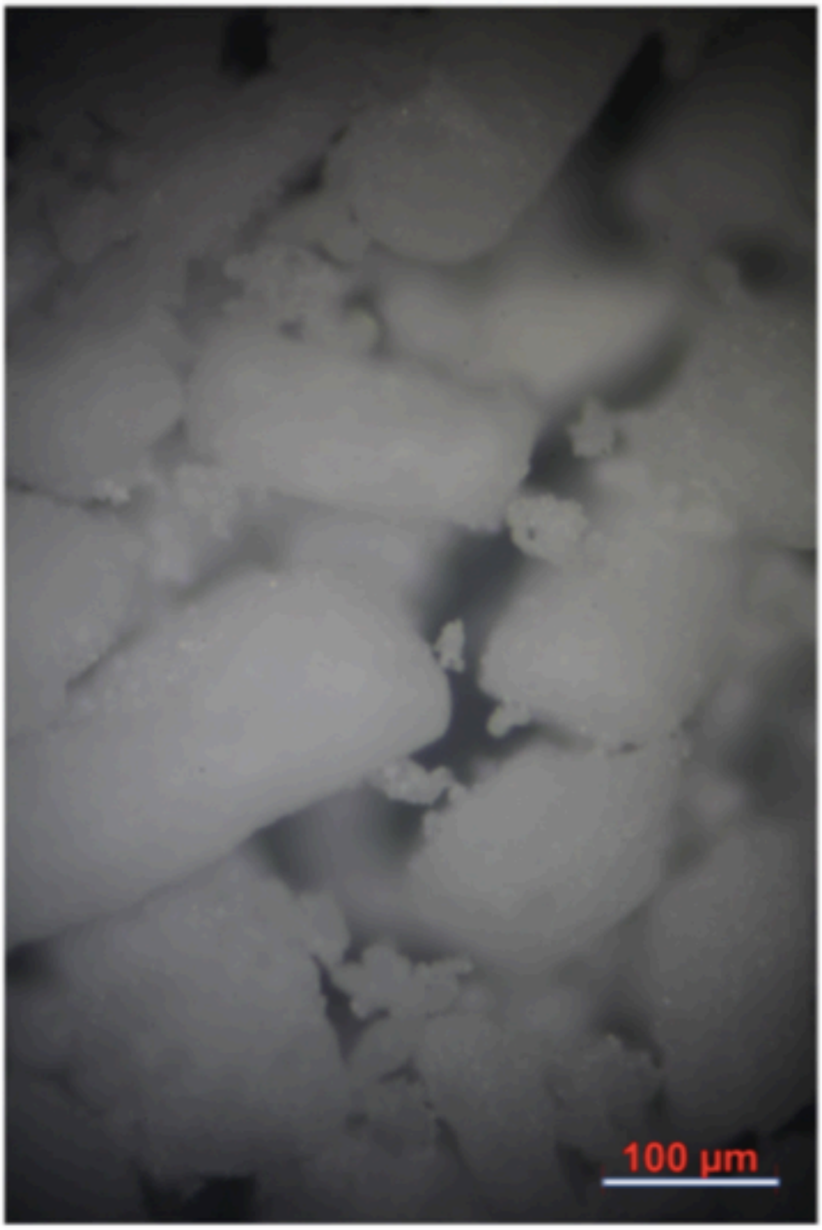

Fig. 1: Optical image of anhydrate ectoine sample.

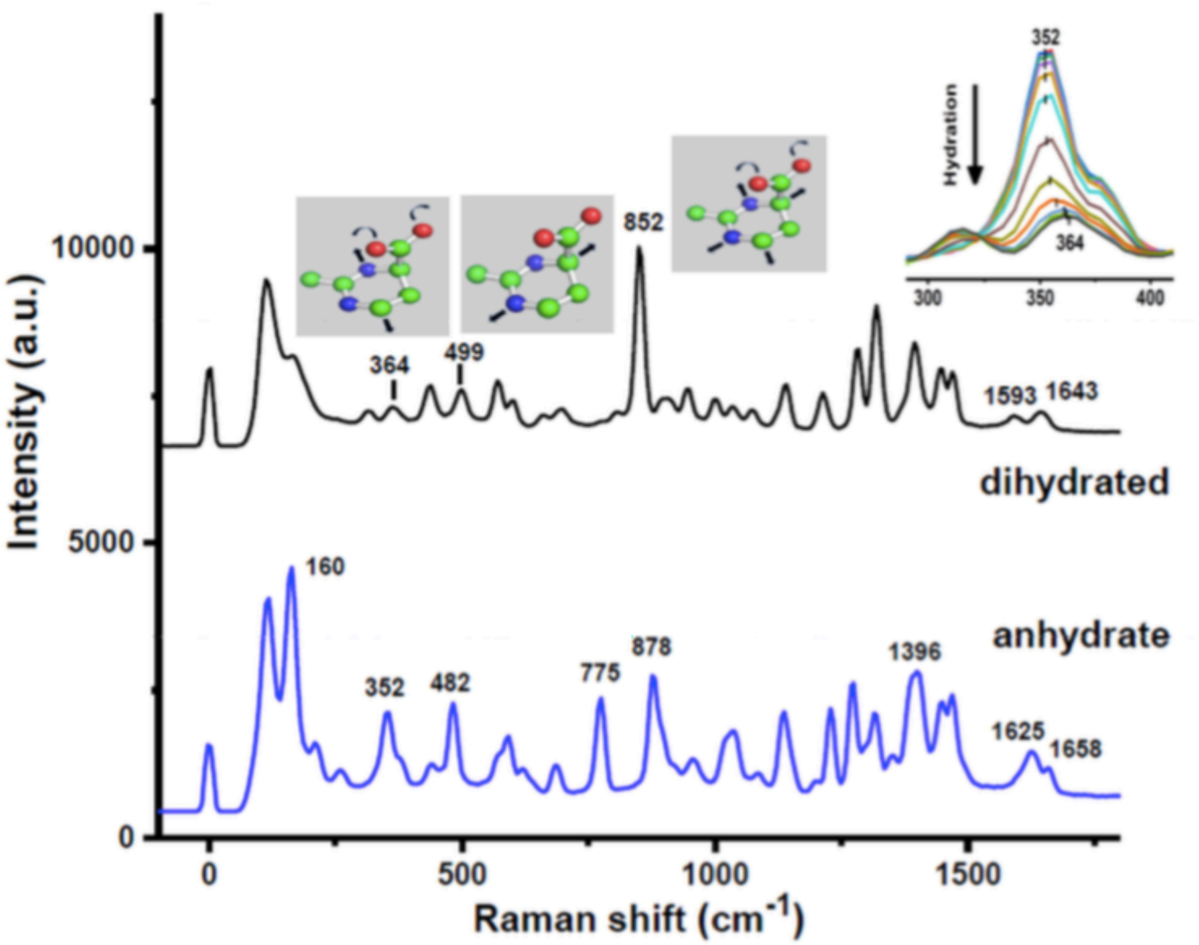

Fig. 2: Raman spectra of anhydrate and dihydrate ectoine. The insets depict the ring deformation modes involved in Fermi resonance and the shift of one of the deformation modes upon hydration.
